# Erwartete Effekte der neuen Weiterbildungsordnung in der Allgemein- und Viszeralchirurgie: Eine Umfrage unter bayerischen Chirurg*innen und Weiterbildungsassistent*innen

**DOI:** 10.1007/s00104-022-01738-0

**Published:** 2022-10-19

**Authors:** Josefine Schardey, Tobias Huber, Alina Sophie Kappenberger, Fabian Horné, Nicola Beger, Maximilian Weniger, Jens Werner, Florian Kühn, Ulrich Wirth

**Affiliations:** 1 Klinik für Allgemein‑, Viszeral- und Transplantationschirurige, Ludwig-Maximilians-Universität, Marchioninistr. 15, 81377 Munich, Deutschland; 2 Chirurgische Arbeitsgemeinschaft Junge Chirurgen (CAJC), Deutsche Gesellschaft für Allgemein- und Viszeralchirurgie (DGAV), Berlin, Deutschland; 3 Abteilung für Allgemein‑, Viszeral- und Transplantationschirurige, Universitätsmedizin der Johannes Gutenberg-Universität, Mainz, Deutschland

**Keywords:** Viszeralchirurgie, Chirurgische Weiterbildung, Kompetenzbasierte Ausbildung, Umfrage, Ärzte in Weiterbildung, Visceral surgery, Surgical training, Competency-based education, Survey, Residency

## Abstract

**Einleitung:**

Die neue, vermehrt kompetenzbasierte Weiterbildungsordnung für die chirurgische Weiterbildung (WBO) trat in Bayern im August 2022 in Kraft.

**Methoden:**

Von Mai bis Juli 2022 führten wir eine anonymisierte Onlineumfrage unter den bayerischen Allgemein- und Viszeralchirurginnen und -cChirurgen sowie den Ärzt*innen in Weiterbildung (ÄiW) durch. Ziel war die Erfragung der Erwartungen an die Effekte der neuen WBO.

**Ergebnisse:**

Die Rücklaufquote betrug 35 %; insgesamt konnten Daten von 80 Personen erhoben werden: 36 ÄiW (45 %), 30 Fach- und Oberärzt*innen (37,5 %) und 14 Chefärzt*innen (17,5 %). Die Mehrheit der Befragten arbeitete an einem Universitätsklinikum (38,8 %) oder Regelversorger (35 %). Eine Stärkung der Handlungskompetenz durch Umsetzung der neuen WBO erwarten 41,3 % und 55,7 % sehen als Ziel ein „selbstständiges Operieren unter teilweiser Aufsicht durch den Ausbilder“. 50 % sehen die geforderten Richtzahlen als nicht erreichbar an, bzw. 55,1 % verneinen ein Erreichen derselben im Zeitraum von 6 Jahren. Etwa 60 % erwarten, nicht die gleiche Anzahl an ÄiW in der gleichen Zeit ausbilden zu können. Fast 75 % der Befragten geben an, dass aus ihrer Sicht eine gute Weiterbildung mit Erreichen einer soliden Handlungskompetenz ohne Überstunden nicht funktioniere. Etwa 44 % der Befragten erwarten, dass die volle Weiterbildung an ihrem Haus auch weiterhin möglich sei.

**Schlussfolgerung:**

Sowohl unter den Weiterbilder*innen als auch unter den ÄiW besteht tendenziell die Sorge, dass eine realistische Weiterbildung – insbesondere das Erreichen der Richtzahlen in der bisher üblichen Weiterbildungszeit – nicht möglich sein wird. Notwendig ist daher die konsequente Umsetzung einer strukturierten Weiterbildung mit hoher Transparenz der Ausbildung.

## Abstract (en)

**Introduction:**

The new competency-based continuing education regulations for surgical training (WBO) came into effect in Bavaria in August 2022.

**Methods:**

From May to July 2022, we conducted an anonymized online survey among Bavarian general and visceral surgeons and surgical residents (ÄiW). The aim was to survey expectations of the effects of the new WBO.

**Results:**

The response rate was 35%. In total data could be collected from 80 persons, 36 ÄiW (45%), 30 specialists and senior physicians (37.5%) and 14 chief physicians (17.5%). The majority of respondents worked at a university hospital (38.8%) or a regular provider (35%). A strengthening of the competence to act through implementation of the new WBO is seen by 41.3% and 55.7% see independent operating under partial supervision by the instructor as a goal. Of the respondents 50% see the required case numbers as not achievable and 55.1% deny reaching them in the expected period of 6 years. About 60% do not expect to be able to train the same number of ÄiWs in the same amount of time. Almost 75% of the respondents state that from their point of view, a good continuing education with the achievement of a solid competence to act would not work without overtime hours. About 44% of the respondents expect that a full surgical training would continue to be possible at their institution.

**Conclusion:**

Both among the instructors and among the trainees there is a tendency to fear that realistic training, in particular the achievement of the guideline figures, will no longer be possible in the usual further training time. This necessitates the consistent implementation of structured continuing education with a high degree of transparency in training.

## Hintergrund und Fragestellung

In Anbetracht der Tatsache, dass bis zu der Hälfte aller schwerwiegenden Komplikationen bei chirurgischen Eingriffen potenziell vermeidbar wären [11], liegt es in unser aller Interesse, sicherzustellen, dass Chirurg*innen im Rahmen der Facharztweiterbildung tatsächlich zu kompetenten Fachleuten mit technischen und nichttechnischen Fähigkeiten ausgebildet werden [1]. Bei der bisherigen chirurgischen Ausbildung besteht die Annahme, dass der Kontakt mit Patienten und die Erfahrung über einen bestimmten Zeitraum ausreichen, um die Ausbildung kompetenter Chirurgen*innen zu gewährleisten [13, 16]. Der Schwerpunkt liegt auf dem Zeitaufwand und der Anzahl der durchgeführten Eingriffe [12, 15].

Der 80. Bayerische Ärztetag hat im Oktober 2021 der Neuformulierung der Weiterbildungsordnung (WBO) zugestimmt [4]. Diese geht auf den Beschluss der Muster-WBO auf dem Deutschen Ärztetag 2018 in Erfurt zurück. Dem waren 6 Jahre Gremienarbeit auf Bundesebene unter Beteiligung von Fachgesellschaften und Berufsverbänden vorausgegangen mit dem Ziel, die WBO zu einer kompetenzbasierten Weiterbildung zu entwickeln [4]. Die chirurgische Ausbildung hat sich weg von der Zählung der Verfahren („wie viele haben Sie operiert?“) zur Bewertung der Kompetenz („was können Sie selbständig tun und wie gut?“) entwickelt [16]. Diese Denkweise wird als kompetenzbasiertes Modell bezeichnet [16]. Dies wird bereits in zahlreiche Ländern für die Weiterbildung eingesetzt [10]. In Tab. 1 sind die Handlungskompetenzen dargestellt sowie die Richtzahlen im Vergleich zur WBO von 2004 aufgeschlüsselt. Für die kognitiven und Kenntniskompetenzen verweisen wir auf die Onlinefassung. Obwohl die Gesamtzahl der Eingriffe mit 400 etwa der abzulösenden Weiterbildungsordnung entspricht, wird nun eine genauere Subspezifizierung der Eingriffe verlangt ([3]; Tab. 1).

**Table 1:** 

Alte WBO	Richtzahl Alt	Neue WBO Handlungskompetenz – Erfahrungen und Fertigkeiten	Richtzahl Neu
*Diagnostische Verfahren*			
–	–	Ösophagoduodenoskopien	50
–	–	Koloskopie	50
Durchführung und Befundung von Rekto‑/Sigmoidoskopien	50	Rektosigmoidoskopie	50
–	–	Proktoskopie	50
Notfall und Intensivmedizin	–	–	–
Punktionen und Legen von Drainagen	10	–	k. A.
Zentralvenöse Zugänge	25	–	20
Thoraxdrainage	10	–	k. A.
Infusion/Transfusion/Ernährung/Sondentechnik	50	–	k. A.
*Eingriffe an der Bauch- und Bauchwand*			
An Bauchwand und Bauchhöhle einschließlich Resektionen, Übernähungen, Exstirpationen, endoskopischer und interventioneller Techniken, z. B. Lymphknotenexstirpationen, Entfernung von Weichteilgeschwülsten, explorative Laparotomie, Magen‑, Dünndarm- und Dickdarmresektionen, Notversorgung von Leber- und Milzverletzungen, Appendektomie, Anus-praeter-Anlage, Hämorrhoidektomie, periproktitische Abszessspaltung, Fistel- und Fissurversorgung, *davon*	400	–	Ca. 400
–	–	Explorative Laparotomie/-skopie	30
–	–	Laparotomien und deren Verschluss	50
–	–	Laparoskopien	50
–	–	Explorative Laparotomie/-skopie	30
Appendektomien	20	–	20
Cholezystektomien	25	–	35
–	–	Magenteilresektion	3
Adhäsiolysen	10	Komplexe Adhäsiolysen	10
Dünndarmresektionen	10	–	10
Dickdarmresektionen	10	Eingriffe am Kolon	30
–	–	Stoma: Anlage und Rückverlagerung	10
Proktologische Operationen	20	Endarmoperation	10
–	–	Hämorrhoidenoperation einschließlich Therapie einer Fissur	20
Herniotomien	25	Operative Therapie von Hernien, auch minimal-invasiv, davon:	–
–	–	Leistenhernie	40
–	–	Bauchwandhernie	10
–	–	Narbenhernie	10
*Schilddrüse/Halseingriffe*			
Operative Eingriffe an Kopf/Hals, z. B. Schilddrüsenresektionen, Tracheotomien	25	Zervikale Eingriffe z. B. Tracheotomie, Lymphknotenprobeexzision	k. A.
–	–	Eingriffe an der Schilddrüse, davon	25
–	–	Schilddrüsenresektionen	20

Da die neue Weiterbildungsordnung in Bayern im August 2022 in Kraft tritt, führten wir zusammen mit der Chirurgischen Arbeitsgemeinschaft Junge Chirurgie (CAJC) der Deutschen Gesellschaft für Allgemein- und Viszeralchirurgie (DGAV) eine Befragung unter den bayerischen Chirurginnen und Chirurgen sowie den Ärzt*innen in Weiterbildung (ÄiW) im Fach Allgemein- und Viszeralchirurgie zu den zu erwartenden Effekten auf die chirurgische Weiterbildung durch. Zudem sollte untersucht werden, ob sich die Erwartungen der „Jungen Chirurgie“ von denen der „Ausbilder*innen“ unterscheiden.

## Studiendesign und Untersuchungsmethoden

### Rekrutierung

**Figure 1:**
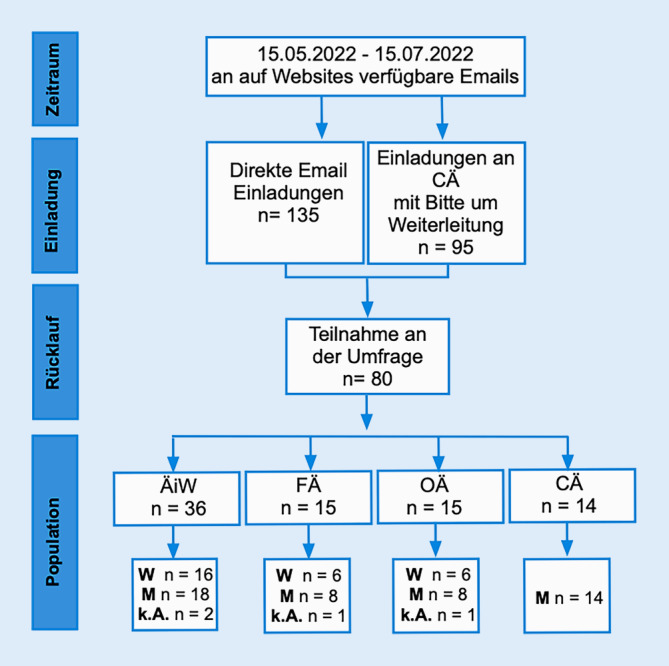

Die anonymisierte Umfrage wurde von Mai bis Juli 2022 durchgeführt (Abb. 1). Da die bayerische Landesärztekammer kein Verzeichnis der ÄiW in der Allgemein- und/oder Viszeralchirurgie führt, wurden die bayerischen Weiterbildungsassistent*innen über den/die jeweilige/n Chefarzt oder Chefärztin mit der Bitte um Weiterleitung an die Mitarbeiter kontaktiert.

### Umfrage

Als Umfragetool diente die Software evasys (evasys GmbH, Deutschland). Die Studie wurde von der Ethikkommission der LMU genehmigt (EK22-0478). Da nicht davon ausgegangen werden konnte, dass die Teilnehmenden genau über die bevorstehenden Änderungen der WBO informiert waren, wurde zunächst ein tabellarischer Überblick über die alten und neuen Richtzahlen der neuen WBO gegeben (vgl. Onlinematerial). Im Anschluss folgten unmittelbar Fragen zu dem Gelesenen in skalierter Form, offene Fragen sowie allgemeine personenbezogene Fragen. Der Fragebogen der Studie ist online unter https://www.scientific-surveys.uni-muenchen.de/evasys/online.php?p=Chirurgie zu finden.

### Statistik

Es wurde von einer erreichbaren Population von 500 Ärzt*innen ausgegangen, für die die neue WBO von Relevanz ist, was bei einem 95 %-Konfidenzniveau und einer Fehlerquote von 10 % bei Meinungsumfragen einer erforderlichen Stichprobe von *n* = 80 entspricht. Die Analyse der Daten erfolgte mittels SPSS Version 25 (IBM, USA) und mittels Graph-Pad Prism 9 (GraphPad Software Inc., USA). Je nach Größe der Stichprobe wurde eine deskriptive Statistik mittels des χ^2^-Test oder Fisher’s exact test durchgeführt; *p*-Werte < 0,05 wurden als signifikant angesehen.

## Ergebnisse

### Rücklaufquote

Die erforderliche Stichprobe von 80 Teilnehmern wurde erreicht, was bei 230 versendeten E‑Mail-Einladungen einer Rücklaufquote von 35 % entspricht (Abb. 1). Bezogen auf die Gesamtpopulation der Zielgruppe bei 2021 in Bayern gemeldeten 692 Chirurg*innen für Allgemein- und Viszeralchirurgie beträgt der Anteil der Population der Fachärztinnen und Fachärzte insgesamt 6,35 %. Durch die BLÄK wird die Anzahl der ÄiW in Bayern nicht erfasst, jedoch wurden im Jahr 2021 bei 47 Personen Facharztprüfungen durchgeführt (E-Mail-Anfrage BLÄK 06.05.2022), somit lassen sich mindestens 300 ÄiW über 6 Weiterbildungsjahre nur schätzen, was einer Teilnahme von ca. 12 % der ÄiW ergibt. Von den 95 angeschriebenen Chefärzt*innen antworteten 14, sodass hier die Rücklaufquote 15 % beträgt.

### Demografische Daten

**Figure 2:**
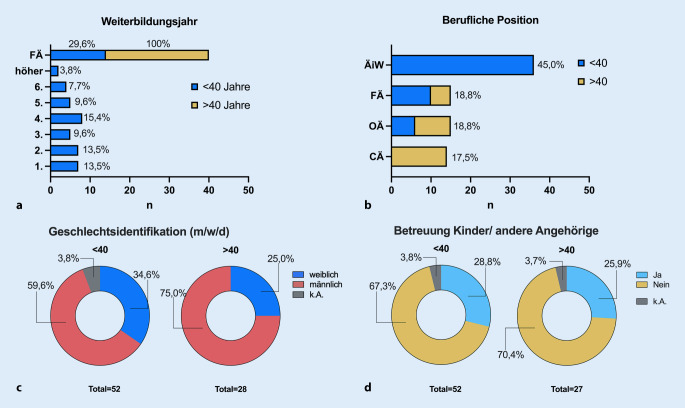

Alle befragten Personen über 40 Jahre waren bereits Fachärzt*innen (FÄ), sodass hier die Grenze zwischen „Junger Chirurgie“ und „Weiterbilder*innen“ gezogen wurde, von den Personen < 40 Jahren waren nur knapp 30 % FÄ (Abb. 2a). 45 % der Befragten waren ÄiW; diese wiesen alle ein Alter unter 40 Jahren auf (Abb. 2b). Der Anteil der > 40-Jährigen stieg mit höherer beruflicher Position, hiervon waren 50 % der Teilnehmenden Chefärzte (Abb. 2b). Bei den < 40-Jährigen betrug die Geschlechtsverteilung m:w bereits 60 % zu 35 %, wohingegen die Chirurg*innen > 40 Jahre 25 % ausmachten (Abb. 2c). Die Betreuung von Kindern oder Angehörigen war in beiden Altersgruppen etwa gleich verteilt (28,8 % vs. 25,9 % der unter und über 40-Jährigen, vgl. Abb. 2d). Insgesamt arbeiten 96 % der Befragten in Vollzeit.

### Arbeitsstelle

**Figure 3:**
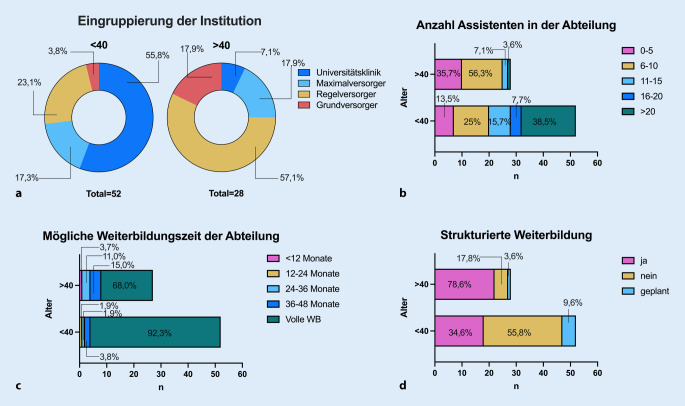

Die Mehrheit der Teilnehmer*innen arbeitet in einem Universitätsklinikum (38,8 %) oder Regelversorger (35 %), wobei die < 40-Jährigen signifikant häufiger an einem Universitätsklinikum arbeiten und die > 40-Jährigen an einem Regelversorger (χ^2^; *p* < 0,001, Abb. 3a). Insgesamt waren 26,5 % der Teilnehmenden in Abteilungen mit mehr als 20 ÄiW tätig. Der überwiegende Anteil (56,3 %) stammte aus Abteilungen mit bis zu 10 ÄiW, wobei sich die Gruppe der < 40-Jährigen am häufigsten in Abteilungen mit > 20 ÄiW befanden (38,5 %), (Abb. 3b), hier unterschied sich die Verteilung der Abteilungen der über und unter 40-Jährigen in der Zusammensetzung signifikant (χ^2^; *p* < 0,001, Abb. 3b). Die < 40-Jährigen arbeiteten zu 92,3 % in Abteilungen, in denen die volle Weiterbildung möglich ist, in den Abteilungen der > 40-Jährigen war das immerhin noch zu 68 % möglich, (χ^2^; *p* = 0,056, Abb. 3c). Eine strukturierte Weiterbildung in der Abteilung wird von lediglich 34,6 % der < 40-Jährigen angegeben, diese sei jedoch in etwa 10 % der Fälle geplant. Die Teilnehmer > 40 Jahre geben dagegen in fast 79 % eine strukturierte Weiterbildung an (χ^2^; *p* < 0,001, Abb. 3d).

### Erwartungen an die neue Weiterbildungsordnung

**Figure 4:**
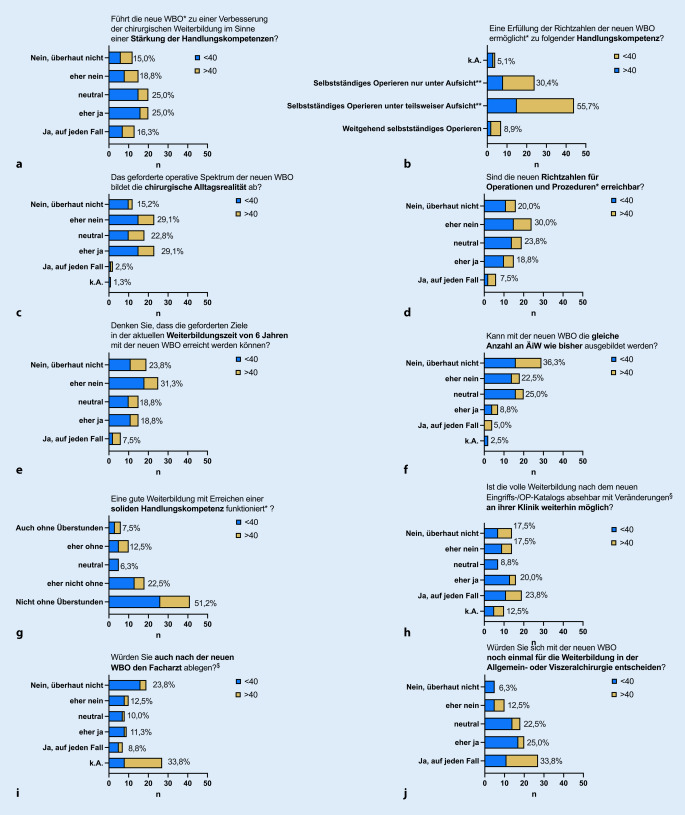

Die Erwartungen (bei skalierten Fragen zusammengenommen: ja/eher ja vs. nein/eher nein) an die neue Weiterbildungsordnung divergierten nicht signifikant zwischen den Altersgruppen (χ^2^; *p* > 0,05), sodass diese im Folgenden in einer Abbildung dargestellt sind (Abb. 4).

Eine Stärkung der Handlungskompetenz durch Umsetzung der neuen WBO sehen immerhin 41,3 % der Befragten (Abb. 4a), 55,7 % erwarten das Ziel eines „selbstständigen Operierens unter teilweiser Aufsicht durch den Ausbilder“ (Abb. 4b). Die chirurgische Alltagsrealität sieht nur knapp ein Drittel der Befragten durch das geforderte operative Spektrum der neuen WBO abgebildet (Abb. 4c), die geforderten neuen Richtzahlen sehen 50 % der Befragten als nicht bzw. eher nicht erreichbar an (Abb. 4d). 55,1 % verneinen ein Erreichen der Richtzahlen im Zeitraum von 6 Jahren, nur etwa ein Viertel hält dies für möglich (Abb. 4e). Passend hierzu erwarten fast 60 %, nicht mehr die gleiche Anzahl an ÄiW in der gleichen Zeit ausbilden zu können (Abb. 4f). Überraschende Einigkeit herrschte in Bezug auf Überstunden: fast drei Viertel der Befragten geben an, dass aus ihrer Sicht eine gute Weiterbildung mit Erreichen einer soliden Handlungskompetenz ohne Überstunden nicht/eher nicht funktioniere (Abb. 4g).

Etwa 44 % der Befragten erwarten, dass die volle Weiterbildung an ihrem Haus mit der neuen WBO ggf. mit Veränderungen auch weiterhin möglich sei (Abb. 4h).

Da theoretisch die Facharztprüfungen für ÄiW, die vor dem 01.08.2022 ihre Weiterbildung beginnen, auch nach der neuen WBO abgelegt werden könnte, wurden die Probanden hiernach befragt: Etwa 20 % der Befragten gaben an, dass sie theoretisch die Facharztprüfung auch unter den Anforderungen der neuen WBO ablegen würden (Abb. 4i). Die Mehrheit der Befragten würde sich auch mit der neuen WBO wieder für die Ausbildung zum Facharzt für Allgemein- und Viszeralchirurgie entscheiden (Abb. 4j).

## Diskussion

Unsere Umfrage ergab, dass sowohl unter den Weiterbilder*innen, als auch unter den ÄiW überwiegend die Sorge besteht, dass eine realistische Ausbildung insbesondere das Erreichen der stärker spezifizierten Richtzahlen in der bisher üblichen Weiterbildungszeit nicht mehr möglich ist. Der Einsatz von Überstunden wird von fast 75 % der befragten Weiterbildungsassistent*innen und Ausbilder*innen als notwendig für eine gute Weiterbildung erachtet.

Die Sicherheit der Patient*innen und die Qualität der Versorgung sowie wirtschaftliche Erfordernisse sind ins Zentrum des Interesses von Leistungserbringern, Interessengruppen und der Öffentlichkeit gerückt [16]. Auch die Arbeitszeitregelungen durch das europäische Arbeitszeitgesetz haben die Weiterbildungsprogramme beeinflusst [6]. Es ist zudem davon auszugehen, dass insbesondere technisch anspruchsvolle chirurgische Eingriffe im Ausbildungskatalog im klinischen Alltag immer schwieriger umsetzbar werden [6]. Das übergeordnete Ziel ist es, eine Ausbildung zu erhalten, die den Chirurginnen und Chirurgen Kenntnisse und Fähigkeiten für ihre künftigen Tätigkeitsbereiche vermittelt [16]. Diese geforderten Fähigkeiten in einem vereinheitlichten Programm für alle chirurgisch Tätigen zu erreichen ist schwierig, da sich hier mitunter auch interindividuelle Unterschiede der ÄiW ergeben können. In der chirurgischen Ausbildung ist daher zu Recht ein Übergang von der Zahl der Verfahren zur kompetenzbasierten Ausbildung erforderlich. Diese unterlag bisher jedoch keiner Qualitätskontrolle, die fortschreitende Einführung des kontinuierlich geführten eLogbuchs [7] bieten sich hier Chancen für die Zukunft.

Eine abgestufte Übertragung von Verantwortung und Selbständigkeit sollte logischen und schrittweisen Pfaden folgen, um die entsprechende Kompetenz zu erreichen, wie beispielsweise dem Teilschrittkonzept [2, 17]. Eine aktuelle multizentrische Umfrage untersuchte die Durchführung des Teilschrittkonzepts in 21 operativen Zentren [17]: Die Zahl der Weiterbildungseingriffe und Teilschritte der ÄiW wurde über- und die Zahl der tatsächlich durchgeführten Teilschritte unterschätzt. Die Daten der Befragung deuteten auf eine geringe Beteiligung von ÄiW im Operationssaal hin, dem Teilschrittkonzept wurde jedoch eine hohe Bedeutung für die chirurgische Ausbildung zugestanden [17].

Durch Mindestmengen und Zertifizierungen könnte es überdies zu einer weiteren Verknappung bestimmter Operationen als Ausbildungseingriffe kommen. Durch eine zunehmende Spezialisierung kommt es zu einer Akkumulation bestimmter Eingriffe, sodass eine vollumfängliche Weiterbildung gegebenenfalls in einem regionalen Netzwerk an Kliniken verschiedener Versorgungsstufen ermöglicht werden kann. Dies unterstreicht die Erfordernis konzeptioneller Änderungen und einer Kontrolle der chirurgischen Ausbildung [17]. Eine weitere Möglichkeit, dem entgegenzuwirken, wäre es, Eingriffe zu simulieren, um die erworbenen Fähigkeiten im operativen Setting gezielter umsetzen zu können. Das Simulationstraining bietet die Möglichkeit, ein breites Spektrum an Eingriffen und Verfahren zu üben [6]. Eine ebenfalls kürzlich veröffentlichte deutschlandweit durchgeführte Umfrage ergab, dass überhaupt nur 35 % der deutschen Kliniken über Simulatoren verfügen, wobei hochwertige Simulatoren an Universitätskliniken am häufigsten vertreten waren [6].

Eine gute, strukturierte Weiterbildung führt auch zu größerer Zufriedenheit der ÄiW [8]. Eine groß angelegte Umfrage unter 729 ÄiW bereits aus dem Jahr 2010 konnte die wichtigsten Punkte berechnen, welche Verbesserungspotenziale für Arbeitsplatzzufriedenheit und -bindung der ÄiW darstellen: 1. Strukturierung der Weiterbildung, 2. Prozessoptimierung im Alltagsgeschäft, 3. Verfestigung sozialer Netzwerke, 4. Feedbacksysteme, 5. Abwechslung und Verantwortungsübernahme im Alltag [8]. Ein von der CAJC erarbeitetes Modell für eine „lebenswerte Chirurgie“ zur Gewinnung und Halten des chirurgischen Nachwuchses ist dem ganz ähnlich [14]: Es zeichnet sich durch die Verzahnung von Vereinbarkeit von Familie und Beruf/Work-Live-Balance, einer besseren Planung des Klinikalltags, einer positiven Feedbackkultur sowie neuen Arbeits(zeit)modellen, mehr Zeit für Lehre und Forschung mit dem oben genannten Teilschrittkonzept in Verbindung mit einem festen Weiterbildungskurrikulum, der Führung eines Logbuches, regelmäßigen Weiterbildungsgesprächen, interklinischen Übungsmöglichkeiten und der Freistellung für Weiterbildungsmaßnahmen aus [14]. Darüber hinaus gilt es, delegierbare ärztliche Tätigkeiten auch an entsprechend geschultes und vorgehaltenes Personal tatsächlich zu übertragen, um die Weiterbildungszeit sinnvoll für operative und klinische Tätigkeiten zu nutzen [5].

Über die signifikant unterschiedlichen Angaben bezüglich des Vorhandenseins einer strukturierten Weiterbildung zwischen „Junger Chirurgie“, die überwiegend an Universitätsklinika beschäftigt ist, und den „Weiterbilder*innen“, die überwiegend an Häusern der Regel- und Maximalversorgung beschäftigt sind, kann nur spekuliert werden. Dies könnte einerseits durch unterschiedliche Perspektiven erklärt werden, was genau als strukturierte Weiterbildung verstanden wird. Andererseits könnte dieses Konzept in anderen Versorgungsstufen bereits vermehrt umgesetzt werden, um den Standort attraktiver zu machen.

Der hohe Anteil der an in dieser Studie teilnehmenden Chefärzte, die in den Abteilungen häufig die Weiterbildungsbefugnis haben, unterstreicht die Bedeutung dieses Themas. Dass die strukturierte pädagogische Ausbildung als Kernaufgabe für Chirurginnen und Chirurgen dringend erforderlich ist, zeigen die zahlreichen „Train-the-Trainer“-Initiativen [9]. Für die Weiterbilder*innen ist es überdies wichtig, dass die Weiterbildungsbefugnis für die neue Weiterbildungsordnung zeitnah beantragt werden muss.

### Limitationen

Die Onlineumfrage erreichte eine Rücklaufquote von ca. 35 % der versendeten E‑Mail-Einladungen, wobei diese vermutlich in Realität deutlich niedriger angesiedelt ist, da die genaue Anzahl an Weiterverbreitungen durch E‑Mail-Weiterleitungen unklar ist. Weiterhin wurden nur Kolleginnen und Kollegen angeschrieben, bei denen E‑Mail-Adressen zumindest der Sekretariate oder direkt über die Klinikwebsite verfügbar waren. Dies war bei Universitätskliniken häufiger der Fall, was zu einer Unterrepräsentierung der Grund- und Regelversorger führen könnte.

Da die Weiterbildung Sache der Länder ist, wurde diese Umfrage nur unter bayerischen Chirurg*innen sowie ÄiW durchgeführt. Die Anzahl der Weiterbildungsassistenten wird nach wie vor nicht von der Ärztekammer erfasst. Auch hier bietet das eLogbuch eine gute Möglichkeit zu mehr Transparenz [7]. Mit im vergangenen Jahr immerhin 47 neuen Fachärzt*innen für Viszeralchirurgie kann man, einen gewissen Verlust von Abbrüchen eingerechnet, auf ca. 300 ÄiW in Bayern im Fach Allgemein- und Viszeralchirurgie schließen. Somit ergibt sich trotzdem noch eine ca. 12 %ige Teilnahmerate bei den ÄiW und knapp 15 %ige Teilnahmequote der angeschriebenen Chefärzte. Mit 80 Teilnehmern kann diese Umfrage als repräsentativ für die Zielgruppe in Bayern gelten. Die Musterweiterbildungsordnung wurde in den meisten Landesärztekammern weitestgehend übernommen [18], sodass die erwarteten Effekte auch auf andere Bundesländer übertragbar sein sollten.

Insgesamt ist diese Meinungsumfrage eher abstrakt, da zunächst die Information durch Vergleichen geänderter Richtzahlen eingeholt werden musste. Dies könnte möglicherweise abgeschreckt und zu einer eher geringen realen Rücklaufquote geführt haben. Eine doppelte Beantwortung der Umfrage war theoretisch möglich. Es wurden keine Fragen zur kognitiven und Methodenkompetenz gestellt, obwohl diese ebenfalls neu eingeführt wurden, da in der neuen WBO hierzu weitere Ausführungen fehlen. Antwortverzerrungen sind als eher gering einzuschätzen, da der Großteil der Fragen zur Einschätzung der Auswirkungen der Effekte der neuen WBO in skalierter Form gestellt wurde.

Zusammengefasst besteht aus Sicht der Autoren die Möglichkeit einer Einforderung konkreterer Zahlen im Sinne einer besseren Ausbildung durch erhöhte Transparenz (Stichwort: eLogbuch), was bei konsequenter Umsetzung mit einer höheren operativen Handlungskompetenz einhergehen könnte. Andererseits besteht jedoch die begründete Gefahr einer möglichen Verlängerung der Weiterbildungszeit durch logistische Probleme bei einzelnen Anforderungen wie etwa der endoskopischen Untersuchungen. Diese können in manchen Kliniken vermutlich nur durch in- oder externe Rotation in eine Endoskopieeinheit oder -klinik ermöglicht werden, womit diese spezifischen Eingriffe zum Nadelöhr werden könnten. Leider bleibt die Art des Erwerbs der sog. kognitiven und Kenntniskompetenzen offen; hier erfolgen keine Ausführungen zu Fortbildungen, Kursen oder implementiertem Simulationstraining.

## Fazit für die Praxis

- Die Spezifizierung der Richtzahlen könnte zu einer höheren Handlungskompetenz führen.
- Es besteht die Befürchtung, die Richtzahlen seien nicht mehr in der bisher üblichen Weiterbildungszeit erreichbar.
- Eine erhöhte Transparenz der chirurgischen Weiterbildung, z. B. durch das eLogbuch, ist hier flächendeckend notwendig.
